# Toxins, Pathogenicity, Anti-Toxins, a Bicentennial Contribution

**DOI:** 10.3390/toxins16020097

**Published:** 2024-02-10

**Authors:** Michel R. Popoff, Daniel Ladant

**Affiliations:** 1 Unité des Toxines Bactériennes, Institut Pasteur, Université Paris Cité, CNRS UMR 2001 INSERM U1306, F-75015 Paris, France; 2 Unité de Biochimie des Interactions Macromoléculaires, Institut Pasteur, Université Paris Cité, CNRS UMR 3528, F-75015 Paris, France; daniel.ladant@pasteur.fr

The bicentenary of Louis Pasteur’s birth raises the opportunity to revisit the activity and influence of L. Pasteur and collaborators in the field of toxins. Microorganisms have been observed since the 17th century, but L. Pasteur clearly demonstrated their main properties, namely that they are living organisms that disseminate by contamination from an infected site to a sterile one and not by spontaneous generation, and that they induce substrate modification such as fermentation. Thus, he defined the basis of a scientific approach of the then nascent scientific domain, microbiology. L. Pasteur and other pioneers in this emerging field, in particular Robert Koch, discovered that certain microorganisms are responsible for specific diseases. The main objective of L. Pasteur was the prevention of infectious diseases, and he developed the concept of prevention with attenuated microorganisms; he called this concept vaccination in honor of Edward Jenner. The success of prevention of rabies pushed the building of the Institut Pasteur in Paris. This institute was devoted to the treatment of rabies, research on infectious diseases, and training on microorganisms. The first microbiology course in the world, called “microbie technique”, was taught by Emile Roux (1888), who succeeded to L. Pasteur and Emile Duclaux as Director of the Institut Pasteur. Albeit L. Pasteur knew of the existence of toxic soluble factors produced by putrefying bacteria; he was more convinced that pathogenic microorganisms act by depletion of vital substrates [1,2]. Scientists of the Institut Pasteur characterized various bacterial and animal toxins. This Special Issue retraces the main steps of the involvement of scientists of the Institut Pasteur in bacterial and animal toxins.

The history of the discovery of bacterial toxins with the contribution of Pasteurians is detailed in the article by Jean Marc Cavaillon (Contribution 1). Putrefying bacteria were the first to be recognized as producing potent lethal poisons. This was observed by the Danish Peter Ludvig Panum in the middle of the 19th century. The term toxin was introduced by the German professor of medicine Ludwig Brieger who characterized several toxic compounds from putrefaction. In the end of 19th century, Elie Metchnikoff and other Pasteurians in the Institut Pasteur of Paris investigated the poisons produced by putrefying bacteria. One of the first bacterial toxins produced by a pathogenic bacterium was the diphtheria toxin. Alexandre Yersin and E. Roux showed that culture filtrates of *Corynebacterium diphtheria* are lethal for experimental animals and concluded that this pathogen produces a toxin responsible for the symptoms of disease and death. In addition, they found that sublethal injections of culture filtrates induced protection against virulent *C. diphtheria*, supporting the idea that a soluble substance elaborated by the pathogen, and not the entire microbe as previously used for fowl cholera and anthrax vaccination by L. Pasteur and collaborators, is sufficient to induce a protective immunity. This was called “chemical vaccination”. A key step in the preparation of antigens was achieved by Gaston Ramon, a French veterinarian who was in charge of producing horse antisera at the Institut Pasteur. He found that diphtheria toxins treated with formalin were no longer toxic but retained their antigenicity. This was successfully applied to tetanus toxins, and then to other toxins. These toxin antigens were designated anatoxins. G. Ramon also developed the concept of adjuvants of immunity. The contributions of E. Metchnikoff in the discovery of cholera toxin and cholera protection, as well as the deciphering of endotoxins by Richard Pfeifer, Alexandre Besredka, and André Boivin, are introduced in this work. The complementarity between French and German scientists at the dawn of microbiology and immunology closes this historical manuscript.

Investigations into anthrax and its protection were a major milestone in the development of the nascent microbiology and for the notoriety of L. Pasteur. Albeit the causative agent of anthrax, *Bacillus anthracis*, was previously observed by Rayer and Davaine, R. Koch validated the concept that each infectious disease is caused by a specific pathogen. L. Pasteur, in the famous vaccination assay against anthrax at Pouilly Le Fort, acquired a great reputation in promising a fight against infectious diseases. The historical aspects about *B. anthracis* as well as the recent research activities in this field including structural analysis, biochemistry, genetics, bacterial host–cell interactions, in vivo pathogenicity, and therapeutic developments carried out at the Institut Pasteur are reviewed by P. Goossens (Contribution 2).

The article of Camille Locht (Contribution 3) reports the contribution of Pasteurians in the characterization of *Bordetella pertussis* toxins. The agent of whooping cough, *B. pertussis*, was identified and isolated by Jules Bordet and Octave Gengou at the Institut Pasteur of Paris and Bruxelles at the beginning of the 20th century. It appears soon that whooping cough is due to toxic compounds produced by this microorganism. The discovery and characterization of the major toxins, lipo-oligosaccharide, adenylyl cyclase (ACT), and pertussis toxin (PTX) are described. ACT was extensively studied by Pasteurians regarding the regulation of its synthesis, structure-function, and mode of action. ACT binds to calmodulin in a Ca^++^-dependent manner prior to exerting its toxic and hemolytic activity. Notably, ACT induces apoptosis of macrophages. The unique properties of ACT led to multiple applications such as ACT use as a vehicle to deliver epitopes to immune cells, targeting of dendritic cells, and induction of cytotoxic and humoral immune responses, as well as construction of a two-hybrid system based on the two complementary ACT fragments. PTX is a major virulence factor of *B. pertussis* for which scientists of the Institut Pasteur of Lille largely contributed to deciphering the structure/function and mode of action. PTX retains a complex structure constituted of an enzymatic subunit and a hexamer of four distinct cell receptor binding subunits. Better understanding of the enzymatic mechanism (ADP ribosylation) led to the development of novel vaccines: an acellular vaccine based on genetically inactivated PTX and a live attenuated *B. pertussis* vaccine strain which confers the advantage to induce a mucosal immunity. The live vaccine is under clinical development (Contribution 1).

Some toxigenic bacteria are responsible for respiratory diseases and have been investigated by several Pasteurians such as Lhousseine Touqui, Michel Chignard and collaborators (Contribution 4). A host defense mechanism consists of the secretion of phospholipase A2 (PLA2). Albeit secreted PLA2, notably sPLA2-IIA, have a proinflammatory role, they exert a major bactericidal function, especially against Gram-positive bacteria. Alveolar macrophages are one of the main sources of sPLA2-IIA and the expression of this enzyme can be modulated by various bacterial factors including bacterial toxins. The article of L. Touqui and collaborators reviews the modulation of sPLA2-IIA in airway infection and their pathophysiological consequences (Contribution 4). Lipopolysaccharide from Gram-negative bacteria and exotoxin S of *Pseudomonas* upregulate the expression of sPLA2-IIA, while other bacterial toxins such as *Bacillus anthracis* oedema and lethal toxins are downregulators of this enzyme. The role of other bacterial factors on sPLA2-IIA expression and consequences are described.

Mycobacteria were another important topic investigated by Pasteurians. Indeed, it is noteworthy to remind that Albert Calmette and Camille Guérin at the Institut Pasteur of Lille developed the BCG (Bacille Calmette Guérin) for the prevention of human tuberculosis. Among the mycobacterial virulence factors, a toxin, called mycolactone, has been identified in the pathogenicity of *Mycobacterium ulcerans*, which is responsible for Buruli ulcers, a severe human skin disease. The team of Caroline Demangel further investigates the activities of mycolactone, which are reviewed in (Contribution 5). Mycolactone is a polyketide with cytotoxic and immunosuppressive effects. Notably, mycolactone impairs the expression of cytokines and membrane receptors in multiple immune cells. C. Demangel and collaborators contributed to the deciphering of the mode of action of mycolactone. This toxin blocks the Sec61 translocon in an inactive conformation, thus inhibiting protein secretion. In addition to being a virulence factor for *M. ulcerans*, by preventing the host immune responses, mycolactone is a potent immunosuppressor with promising therapeutic applications such as in the treatment of inflammatory diseases (skin inflammation, rheumatoid arthritis, inflammatory pain) as well as in oncology and virology. A shorter synthetic molecule (mini-mycolactone), which is easier to produce than mycolactone and retains anti-inflammatory properties, is under development.

An important bacterial phylum concerns the cyanobacteria which possess the unique property to perform oxygenic photosynthesis. Cyanobacteria are very widespread on the surface of the Earth and prosper in extremely diverse ecological sites contributing to the oxygenation of the atmosphere. They show a wide diversity regarding their morphology, physiology, and genome. Some species produce toxins which are a threat to animals and humans. The review of Muriel Gugger (Contribution 6) summarizes the historical aspects of the cyanobacteria study at the Institut Pasteur and the current activity of the Collection of Cyanobacteria (Contribution 6). It was initiated by Roger Stanier who was professor of microbiology at the University of Berkeley, California. In 1960–1961, he visited the laboratory of Jacques Monod at the Institut Pasteur of Paris. In 1971, he was appointed Director of the Microbial Physiology Unit at the Institut Pasteur until his death in 1982. His numerous works focused on fundamental aspects of microbiology such as comparative biochemistry and evolution of microorganisms including cyanobacteria for which he started the collection of the Institut Pasteur. Then, the successors of R. Stanier and collaborators enriched, and further documented the cyanobacteria collection, which is a reference collection for these microorganisms. A great effort has been made in strain purification and in maintaining alive these bacteria. Genome sequencing allowed to investigate the diversity and phylogeny of the cyanobacteria phylum, as well as to characterize gene clusters involved in the synthesis of cyanotoxins and other natural products. Indeed, cyanobacteria produce numerous unique natural products which can be useful for pharmacological and/or biotechnological applications and developments.

L. Pasteur discovered life without oxygen, and with Jules Joubert he identified the first anaerobic bacterium responsible for disease, a septicemia in sheep. Subsequently the anaerobic bacteriology was developed at the Institut Pasteur the historical aspects of which are reported in the review by M. Popoff and S. Legout (Contribution 7) of this Special Issue. Adrien Veilon amended the method of anaerobic culture and found that many anaerobic bacteria are saprophytes of the oral cavity leading to the distinction of endogenous and exogenous microflora. Severe diseases such as tetanus and botulism were found to be caused by potent toxins produced by specific anaerobic bacteria. Gas gangrenes, which were widespread harmful diseases, were also recognized to be transmitted by other anaerobic bacteria. The production of therapeutic antisera against diseases due to anaerobic bacteria was one of the main activities at the Institut Pasteur of Paris. They took advantage of the improvement of antigen preparation by G. Ramon. Characterization of anaerobes and their toxins was undertaken by Pasteurians. Among them, André-Romain Prévot had a central role in the development of anaerobic bacteriology, notably by investigating the physiology and taxonomy of anaerobes. In the most recent period, important Pasteurian’s contributions, particularly in the team of M. Popoff, have been brought on clostridial toxins including gene sequencing, regulation of synthesis, mode of action, structure/function, and entry into cells.

Among anaerobic bacteria, *Clostridioides difficile* (previously *Clostridium difficile*) has a special place since it is responsible for most nosocomial diarrheas with epidemic evolution in hospitals. Since the emergence of this pathogen in the 1980s, the Institut Pasteur was involved in the identification of *C. difficile* infections and characterization of the causative agent. The team of Bruno Dupuy was interested in the deciphering of the regulation of toxin synthesis which is an important step in the onset of this toxin mediated disease (Contribution 8). The article by B. Dupuy summarizes the major findings in the regulation of toxin synthesis in *C. difficile*. The genes of the major toxins, toxin A (TcdA) and toxin B (TcdB), are located in the chromosomal pathogenicity locus (PaLoc), which also contains two key regulatory genes encoding an alternative sigma factor (TcdR) and an anti-sigma factor (TcdC). Their role was elucidated by B. Dupuy, Linc Sonenshein and collaborators. TcdR is required for the specific transcription of *tcdA* and *tcdB*, while TcdC is a negative regulator. In addition, *C. difficile* toxin synthesis is under the control of a complex regulatory network including general and specific metabolic regulators as well as regulatory loci controlling sporulation, motility, flagellar synthesis, quorum sensing, and SOS response. A comprehensive review of the regulatory genes which have been identified to date, is presented.

Animal toxins are another toxinology field where Pasteurians made a key contribution as reviewed in the article of M. Popoff and colleagues (Contribution 9). The main initial objective was to treat and prevent the envenomations that were a serious threat in certain countries. It was hypothesized that methods used for bacterial toxins could be applied to animal toxins. Cesaire Phisalix, Gabriel Bertrand, and Albert Calmette were pioneers in antivenom serotherapy. They showed that attenuated venoms induce a protective immune response and that hyperimmune sera are efficient therapeutic drugs. Then, Gaston Ramon improved the efficiency of antivenom preparations by using formalin treatment which was successfully developed for bacterial toxins. Thus, the Institut Pasteur of Paris was involved in massive production and distribution of antivenom sera, mainly to Asia and Africa, notably through the international network of the Institut Pasteur. Concomitant characterization of venom composition and mode of action were investigated by Pasteurians. Paul Boquet demonstrated that certain toxins are present in venoms from multiple snake species. His objective was to select the most appropriate antigens to obtain wide polyvalent antisera. P. Boquet investigated the coagulation activity and phospholipases of venoms, and with France Tazieff-Depierre, he analyzed the venom neurotoxic activity. F. Tazieff-Depierre discovered that certain snake toxins bind to acetylcholine receptor and induce paralysis, whereas other toxins from scorpion and sea anemones cause excessive release of acetylcholine and muscle contracture. Cassian Bon who started to work on scorpion venom with F. Tazieff-Depierre, pursued the investigation of neurotoxic activity and phospholipases in snake and scorpion toxins with the perspective to develop therapeutic drugs. More recently, Grazyna Faure continued the exploration of animal phospholipases A2. She notably discovered that a snake toxin modulates certain ion channels opening the way to the development of novel anti-cystic fibrosis agents. In addition to Pasteurians, many French scientists contributed to the exploration of animal toxins.

In the footsteps of L. Pasteur in the deciphering of the origin and transmission of infectious diseases, Pasteurians investigated the mechanisms of pathogenicity. Bacterial and animal toxins were among the first virulence factors that have been identified. Initial concerns were the prevention and treatment of the corresponding diseases and envenomations. Thus, specific toxin-based tools and processes, vaccination and serotherapy, have been developed. As innovative technologies progressed, more and more in-depth characterization of toxins was performed including biochemistry, genetics, structure aspects, enzymatic and cellular activities. Toxins appeared to be not only virulence factors, but also relevant tools to explore physiological cell processes and to develop novel and efficient therapeutics. This Special Issue illustrates the historical aspects of the major Pasteurian contributions to the fascinating story of toxins.

Besides the articles of this Special Issue on Pasteurians and toxins, additional contributions of Pasteurians concern the study of listeriolysins. Indeed, the laboratory of J. Alouf was focused on hemolysins, notably the cholesterol-dependent cytolysin family including listeriolysins. The role of listeriolysins in bacterial invasion was further investigated by the team of Pascal Cossart. The pore-forming toxin listeriolysin O (LLO) is required for *Listeria* escape from phagosome to host cell cytoplasm by disrupting vacuolar membrane [3]. LLO exhibits additional effects contributing to bacterial invasion of cells, notably by remodeling cell surface glycoproteins such as LAMPs (late endosomal membrane protein [4]. Javier Pizarro-Cerda and collaborators demonstrated that the listeriolysin S produced by hypervirulent *Listeria monocytogenes* strains is a membrane associated bacteriocin targeting. Listeriolysin S alters the host gut microbiota and thus facilitates the persistence of *L. monocytogenes* in the intestine and subsequent infection [5,6].
